# A qualitative exploration of the human resource policy implications of voluntary counselling and testing scale-up in Kenya: applying a model for policy analysis

**DOI:** 10.1186/1471-2458-11-812

**Published:** 2011-10-18

**Authors:** Miriam Taegtmeyer, Tim Martineau, Jane H Namwebya, Annrita Ikahu, Carol W Ngare, James Sakwa, David G Lalloo, Sally Theobald

**Affiliations:** 1 Clinical Group, Liverpool School of Tropical Medicine, Liverpool, UK; 2 International Health Group, Liverpool School of Tropical Medicine, Liverpool, UK; 3 Family Health International, Nairobi, Kenya; 4 Counselling Department, Liverpool VCT, Treatment & Care, Nairobi, Kenya; 5 Quality Assurance Coordinator, National AIDS and STD Control Programme, Nairobi, Kenya; 6 Elected Chair, Association of Kenya Medical Laboratory Scientific Officers, Nairobi, Kenya

## Abstract

**Background:**

Kenya experienced rapid scale up of HIV testing and counselling services in government health services from 2001. We set out to examine the human resource policy implications of scaling up HIV testing and counselling in Kenya and to analyse the resultant policy against a recognised theoretical framework of health policy reform (policy analysis triangle).

**Methods:**

Qualitative methods were used to gain in-depth insights from policy makers who shaped scale up. This included 22 in-depth interviews with Voluntary Counselling and Testing (VCT) task force members, critical analysis of 53 sets of minutes and diary notes. We explore points of consensus and conflict amongst policymakers in Kenya and analyse this content to assess who favoured and resisted new policies, how scale up was achieved and the importance of the local context in which scale up occurred.

**Results:**

The scale up of VCT in Kenya had a number of human resource policy implications resulting from the introduction of lay counsellors and their authorisation to conduct rapid HIV testing using newly introduced rapid testing technologies. Our findings indicate that three key groups of actors were critical: laboratory professionals, counselling associations and the Ministry of Health. Strategic alliances between donors, NGOs and these three key groups underpinned the process. The process of reaching consensus required compromise and time commitment but was critical to a unified nationwide approach. Policies around quality assurance were integral in ensuring standardisation of content and approach.

**Conclusion:**

The introduction and scale up of new health service initiatives such as HIV voluntary counselling and testing necessitates changes to existing health systems and modification of entrenched interests around professional counselling and laboratory testing. Our methodological approach enabled exploration of complexities of scale up of HIV testing and counselling in Kenya. We argue that a better understanding of the diverse actors, the context and the process, is required to mitigate risks and maximise impact.

## Background

The expansion of voluntary counselling and testing for HIV (VCT) has implications for human resources. There has been increasing global recognition of the need for task shifting (whereby medical tasks are delegated to less specialised health care workers) in response to the HIV epidemic [1,2]. 2008 saw the launch of new task shifting guidelines [3] with a number of countries reporting the success of this approach in HIV services generally [4,5] and in HIV counselling and testing specifically [6,7]. However, as new cadres of staff emerge, concerns remain over the professional recognition of lay staff, their training and supervisory support as well as quality assurance mechanisms [8,9].

Kenya, like many resource-poor countries, has a chronically under-resourced health care system and a high prevalence of poverty [10]. In the public sector in 2004, there were 3 doctors and 49 nurses per 100 000 population and more than half of all health personnel were based in urban areas [11]. The average health centre has no laboratory staff and a district hospital having an average of only 4 technicians and technologists. Kenya continues to lose large numbers of qualified health care workers, including high numbers of laboratory staff every year [12]. On the one hand the Government of Kenya is committed to scale up of access to HIV services, on the other hand donors are unwilling to fund salaries and a recent International Monetary Fund moratorium on hiring of civil servants means that despite increasing workloads, the government is not able to hire additional health professionals [13,14].

In 2000, when this work commenced, an estimated 2.2 million adult Kenyans were infected with HIV [15]. Prevalence rates showed significant regional and rural/urban variations, average urban prevalence was (10%) nearly twice that in rural areas (5-6%) and some districts in Nyanza province had prevalence rates in excess of 30% [16]. Kenya is an example of a country where task shifting has been employed to facilitate the rapid scale up of client-initiated counselling and testing services in government health facilities [17,18] and high prevalence areas were targeted in the initial phase of scale up. The introduction in 2000 of rapid HIV testing technology that enabled whole blood from a finger prick to be used for testing made it possible for lay counsellors to conduct HIV testing with rapid and reliable results [19]. Kenya then saw a rapid increase in the availability of VCT from 3 sites in 2000 to over 400 registered sites in early 2004 [17].

The Kenyan scenario represents the kind of challenges that health policymakers are faced with in many resource poor countries: the need to increase the flexibility of health services provision balanced with the need for quality and professionalism. New HIV services are provided in a fast-changing context with wide reaching implications for human resources. Kenya has a chronically under-resourced health care system [20]. Staffing implications need to be understood in the context of serious shortages, mal-distribution of staff and financing constraints [1]. We set out to explore the complex interactions between the key actors, the context they operated in and the processes that evolved as human resources policies for the scale up of HIV testing and counselling (HTC) were developed.

## Methods

Qualitative research methodologies were employed, appropriate to exploring policymakers' perceptions and attitudes of human resources for VCT scale up [21,22]. Methods which were carried out simultaneously included participant observation, review of minutes of taskforce meetings and individual in-depth interviews with key informants all conducted by the principal investigator (MT). Scale-up occurred across the country and so the whole of Kenya may therefore be seen as the study area. With the exception of a one week retreat in 2001, all taskforce meetings were held in Nairobi and this is where the policymaker interviews were conducted.

A total of 53 sets of minutes, representing all of the taskforce meetings held between Sept 2000 and July 2004 were reviewed. Close to 80% (42/53) of these had matching diary notes. Consideration was given to the interaction of the individuals as well as the group processes and decisions in analysis of diary notes. MT was thus both a participant and an observer [23], and 'insiders' (co-authors who were members of the VCT taskforce - AI, JHN, CN, JS) as well as 'outsiders' were included in the qualitative analysis process. While the authors' involvement in the process may have led to subjectivity, the close 'insider' involvement allowed unique insights, grounded in a reflexive approach. Awareness of the difficulties, dilemmas and potential biases of the methodology meant that steps were taken to mitigate them. Open-ended questions followed by probes were used in interviews to enable participants' own views to be clearly expressed. The roles of individuals within the group are examined reflexively in the analytical process to assess their influence on policy and in shaping strategies for VCT scale up.

Purposive sampling was employed to recruit interviewees with rich and relevant experience [24]. Interviewees ranged from those with first hand experiences establishing VCT programmes, to technical advisors, donors and Ministry of Health (MoH) officials. Not all were permanent members of the taskforce but all were key political drivers of the scale up process. A total of 22 interviews were conducted, 14 of whom were taskforce members. Interviews were conducted in English by MT between March and May 2004 and were audio-taped with concomitant note-taking. Informed consent was obtained and participants' details were anonymised and coded. However, the gender and position of participants means that many would be able to recognise not only their own inputs but also those of fellow members of the VCT taskforce. Wherever possible (given the time delay) results were sent to participants for feedback and 'participant checking', thus ensuring quality control. Feedback was received from 15 of 22 interviewees (some were no longer contactable), who indicated they were happy with the way their views were identified and portrayed. For these participants, details of gender and location are provided with illustrative quotations; for others these details are not included to maintain confidentiality.

**Figure 1:**
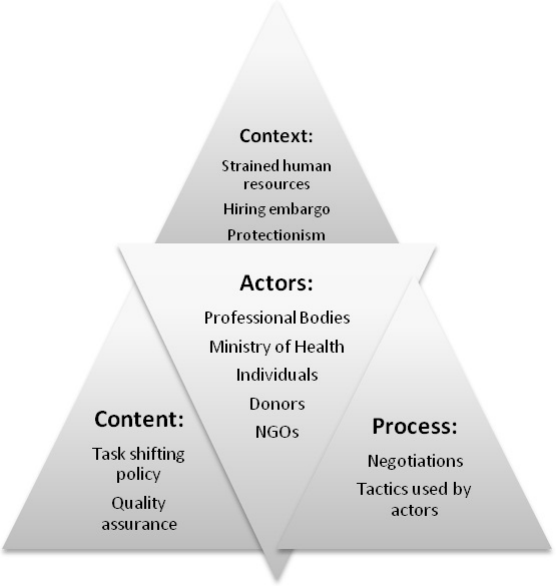
**Policy analysis of VCT scale up (adapted from Walt and Gilson 1994)**.

All 22 interviews were included in the analysis. Data analysis commenced during the data collection period allowing emerging issues to be included in subsequent interviews and issues from participant observation to be included in the interview guide. Once complete, the data were analysed using a thematic framework [25], integrating themes from interviews with those from participant observations and diary notes enabling cross connections to be made. Our analysis was informed by conceptual and empirical work by Walt and Gilson [26,27] and Buse [28], who developed a simplified analytical model, called the 'health policy triangle' (see Figure 1). The model was developed to address the problem that the focus of most policy analysis at the time - in this case related to health reform - was on the content of the policy. Like Reich [29] they believed that policy change is a highly political process and therefore *actors *were at the centre of the process. In addition, attention should be paid to the *processes *of developing policy including how the policy issue arises (agenda setting), how decisions are made in the development and finally how the policy is implemented. Finally, all this takes place with a particular *context *(for example, the particular role of the state, within a specific cultures and the current economic situation), which along with the policy content formed the three apices of the triangle around the actors. This model, or variants of it, continues to be used in resource poor contexts for analysis of health policy [30-32]. In our analysis, too, particular attention was paid to the complex inter-relationships between the actors (forming the centre of the triangle) and the context, processes and content.

All study participants provided written informed consent and were informed of the study by the VCT taskforce at the Kenyan National AIDS and STD Control Programme, which gave explicit permission for meeting minutes to be reviewed. Ethical approval was obtained from the Kenya Medical Research Institute for operational research on the scale up of VCT. This specific study also received institutional approval from the Liverpool School of Tropical Medicine Research Ethics Committee (ref 03.62).

## Results

Data was synthesized from all the different qualitative methods employed, the results shed light on the difficulties faced in instigating task shifting policy. The health policy triangle (see Figure 1) is used to graphically illustrate and structure the findings. The findings are thus presented against the four areas in the triangle: 'context', 'actors', 'process' and 'content'.

### Context of VCT scale up in Kenya

In late 2000 a consultative meeting [19] on VCT set the agenda for scale up, outlined government strategy and reviewed the results of pilot studies conducted in Kenya [33] as well as experience with rapid testing from elsewhere. Key to the discussion was the availability of new rapid testing technologies. These were easy to conduct by lay people, meaning that specialised laboratory training was not necessary, did not require electricity or external reagents and gave results in 15 minutes, thus enabling the giving of results immediately after testing. In 2001 a national taskforce convened to oversee VCT services in Kenya. From the outset it intended to actively promote task shifting such that the burden of extra work created by HIV counselling and testing was distributed throughout the different categories of staff and maximum benefit was gained from the use of lay counsellors. With this in mind a new combined role, designated 'VCT counsellor', was conceived and a unified curriculum for training was published by the National AIDS and STD Control Programme (NASCOP). This covered counselling skills, rapid HIV testing using whole blood and quality assurance [34]. It was open to health care professionals as well as lay counsellors.

Over 2500 VCT counsellors were trained and certified and 900 new sites opened in the following six years. The majority (80%) of these VCT sites were located in government health facilities. At the same time HIV testing in clinical settings was increasing in Kenya with antenatal clinics and hospitals incorporating provider-initiated HIV testing and counselling (PITC) as a routine standard of care. Health facilities trying to find ways to provide HIV counselling and testing in addition to their existing clinical services were experiencing significant new human resource challenges.

### Key actors and their interests in task shifting

Table 1 contains a summary of the key actors and their interests in task shifting. The strategic alliance between donors (who did not support salaries for government staff) and the central Ministry of Health (who were not in a position to hire new staff) was instrumental in encouraging task shifting.

**Table 1:** Key actors and their potential impact on task shifting policy

Actor	Engagement with taskforce	Interest regarding scale up and task shifting policy	Potential support for lay counsellor testing*	Evidence of support or opposition to task shifting
Ministry of Health (central)	Limited but aware	Use existing salary lines: encourage task shifting	+++	Agrees to meetings with all players

Ministry of Health (NACC)	Limited but aware	No significant engagement. Supportive if CBOs and NGOs using lay counsellors	+	National AIDS Strategy sets targets for scale up No significant involvement in the processes

Ministry of Health (NASCOP)	Convened taskforce in 2001 Host meetings	Open as many sites as possible with wide geographic coverage	++	Provides enabling policy environment Open wording to appease all players Accepts variety of financial contributions

Ministry of Health (provincial and district level)	Occasionally invited to meetings	Ensure quality of sites in districts Pragmatic: work within existing resources Maintain good relationships and a motivated workforce	++++	Provides services and input practical advice into policy development Seeks additional donor support

Laboratory associations	Individuals attend meetings on behalf of other actors (e.g. if working for a donor but also an association member). Not formally engaged in taskforce until 2003	Maintain quality of tests Maintain professionalism in testing Create jobs for unemployed laboratory technicians and technologists	-	Engages senior Ministry of Health officials and at times national newspapers to try to prevent task shifting

Counselling associations	Only one (of two possible) counselling associations engaged in taskforce	Recognition for counselling as a profession Maintain quality of counselling Maintain a monopoly on recognised training institutions Continue current curricula on theories of counselling	+	Disagree with length and focus of training curriculum Approach central Ministry of Health directly to request counselling recognised as a cadre in the Ministry

Donors and their implementing partners	Provided driving force for taskforce establishment, including funds for secretariat	No funding for government salaries: encourage task shifting but varying approaches to incentives for lay counsellors Report high client flow Report high numbers of sites opened	+++	Funding to government directly to convene taskforce, collect data etc. Influence through support to implementing partners whose remit is policy change Influence through support to NGO/CBOs

*+indicates support of lay counsellor testing and - indicates lack of support

"We have had a non-replacement policy within the hospitals and within most of the MoH now for years. At this point it is impossible for a major redirection of health personnel into VCT." (Male donor, key informant interview.)

The VCT taskforce was established with donor funding for initial meetings under MoH leadership in late 2000 and was convened by NASCOP. While the backbone of membership was MoH officials, donors and their implementing partners, a number of technical advisors, laboratory and counselling experts with experience in HIV testing and other key stakeholders were invited to attend either from the onset or at various stages over the course of the next few years (seen in the first column of table 1). The inclusion of individuals with experience at the district level allowed human resource lessons, pilot studies and operational research to shape policy development.

Professional associations were also influential, but had a potential to undermine moves towards task shifting. Traditionally, both laboratory and counselling associations have been involved in HIV testing and counselling. Both expressed concerns that scale up and task shifting might compromise quality. The two associations looked at quality from two different angles. Whereas the counselling association did not mind lay counsellors conducting the testing, they were concerned over the length and content of the counselling training. On the other hand, the laboratory association were less concerned with this and more opposed to lay counsellors conducting testing. Laboratory operations are regulated by the Kenya Medical Laboratory Technicians and Technologists Board under an Act of Parliament and personnel may join the Association of Kenya Medical Laboratory Scientific Officers [35] that acted as advocates for its members, rather like a union [36]. Initial taskforce meetings agreeing the VCT guidelines included a number of laboratory professionals who were members, but not official representatives, of the board and association. Like many cadres of health professionals in Kenya there are a significant number of unemployed laboratory staff, as well as an employment embargo, which according to some was imposed by the International Monetary Fund [37,38]. The association did not see the use of task shifting as being in the best interests of it members, the majority of whom saw HIV testing as the prerogative of laboratory technicians and technologists. They referred to the VCT guidelines, which state that they should conduct testing *where available*, to reinforce their arguments.

"it rightly belongs to the lab people to test." (Laboratory technologist, key informant interview.)

The government does not currently recognize the counselling profession as a cadre in the MoH. As well as theory-based university courses in counselling psychology there are many registered counselling organisations. Two large associations of professional counsellors undertake generic training to certificate, higher diploma and Master's level. These organisations saw HIV testing as an opportunity to promote counselling as a profession. They underscored its central role in HIV services and encouraged members to learn how to conduct rapid testing. Once happy with the move to rename the new trainees as 'VCT counsellors' as distinct from 'professional counsellors' the associations shared training curricula with the national committee, took part in the process of developing a unified curriculum and encouraged as many as possible to attend their own or other recognised VCT counsellor training courses, whilst trying to maintain prime position in this market.

A number of large international non-governmental organisations and smaller local organizations, funded by donors, were involved in the provision of VCT services or technical support including procurement, logistics, counselling and testing and social marketing. Some were tasked by their donors to work with government staff, whilst others provided independent services. Donor policies generally restricted direct salary support to government staff but organisations were able to give technical and quality support. A number of the smaller organisations established mechanisms for donor funds to be routed through NGOs most of whom also had a vested interest in sustaining their own funds. The relationship between donors as taskforce drivers who promoted the scale up of lay counsellors and concerns around sustainability were openly debated in Kenya, where donors had high expectations of the speed of scale up and pushed the lay counsellor agenda from early on without formal assessment of longer-term sustainability (see table 1).

### Processes in task shifting

The process of reaching consensus on task shifting sufficient for the publication of unified national guidelines and curriculum involved lengthy discussions. The taskforce took a number of pragmatic early decisions based on the results of a pilot study [39]. They were: to use health professionals selected from all cadres already on the government pay roll, to come up with a uniform training curriculum and to allow non-laboratory staff to conduct testing.

The development of the training manual for VCT counsellors was a contentious issue in the VCT taskforce and between counselling organisations, the MoH, international agencies and technical advisors (*subcommittee minutes 2001*). The development of the manual to the satisfaction of all parties with different views took almost two years to complete. There was disagreement on the length of training, counselling organizations wanted longer training, government agreed to a two-week training and some technical and donor agencies pushed for two-day training on testing alone. The last two groups were concerned that time away from service provision for training would exacerbate existing shortages. There was also disagreement on the content of counselling. The professional counsellors were pushing for a training that was grounded in counselling theories leaving them able to conduct fully client-centred therapeutic sessions. Others argued for an approach that was still client-centred but focused on exploring individual HIV risk issues and others still wanted a simplified training that taught counselling through providing trainees with lists of statements and questions on pre-printed cards that were to be used to guide the sessions. The disparate elements, content, curricula and theories of adult education were brought together in a participatory process and a manual was published in 2002.

Acrimonious debates in the taskforce (*recurring theme in taskforce minutes Nov 2000 - Sept 2003*) dominated the decisions on whether health workers (primarily nurses) or counsellors should provide the counselling and testing. Some committee members argued that it was wrong to divert health workers from clinical duties; others argued that medical diagnoses should remain the prerogative of clinicians and HIV testing the prerogative of laboratory technicians and technologists *(diary notes, March 7-9^th^, 2001; Guidelines Retreat)*. There has been an evolution over time in response to these concerns. Initially health workers provided VCT services delivered in health facilities, and lay or non-medical counsellors provided services in stand-alone or community sites. By 2005 lay counsellors were employed to work as VCT counsellors in health facilities but government funding for these finished in 2009. By 2011, few remained in place. Instead health workers provide HIV testing and counselling as part of 'provider-initiated testing and counselling' (PITC) in health facilities and lay counsellors are employed in stand-alone VCT sites, mobile, outreach and door-to-door HIV testing and counselling programmes. Senior laboratory staff who have been on a training course train and certify VCT counsellors in HIV rapid testing, supervise the VCT counsellors doing testing and support the district medical laboratory in-charges to provide quality assurance mechanisms in VCT sites. In the end, the agreed wording for the guidelines was:

"All HIV testing for VCT should be done by laboratory technologists or technicians. However, in some locations and settings this is not possible. If they have successfully completed an accredited training in testing procedures and are supervised by a laboratory technologist/technician, other health workers may perform simple rapid tests for VCT purposes."

Practically however, only a proportion of testing was conducted by laboratory personnel. A few large stand-alone sites were able to hire one technician or technologist, but most sites, including health facilities, were conducting in-room testing by health care workers *(participant observation, diary notes Nov 21st 2001)*. Some time after VCT was well established in the country, the issue was brought to the attention of the central MoH who tried to placate both sides, changing the wording of the guidelines to add 'or any other person authorised by the Minister of Health'.

"because the government has supported VCT scale up knowing that we don't have enough lab people to cope." (Female programme officer, Nairobi, KII.)

**Figure 2:**
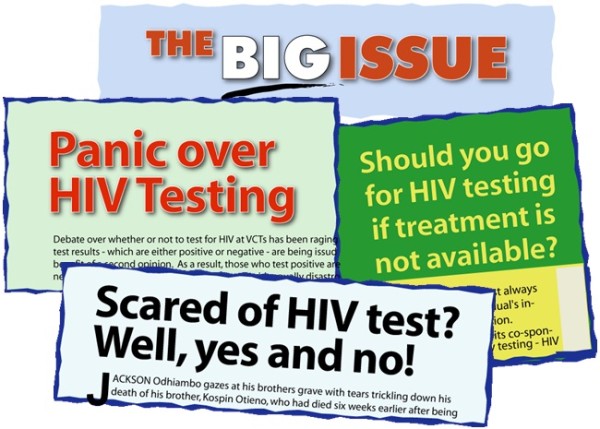
**Sample VCT newspaper headlines August and September 2003**.

At the Annual Conference of Laboratory Scientific Officers in Kakamega in October 2002, data from implementing partners VCT sites were presented that confirmed that lay counsellors who conducted testing were getting a 99.6% level of accuracy according to rigorous external quality assurance [40]. Despite this and due in part to a lack of clear statement from leadership, the dispute continued to rage. Variously described in interviews as a *'turf war' *a *'threat to professionalism' *and *'a concern over quality standards'*, the deliberations over who should conduct testing almost brought VCT scale up to a halt and reached the press in August 2003 with articles raising quality concerns about non-laboratory personnel testing. Popular daily national papers including the Nation, Standard and East African carried almost daily articles on the debate with headings such as 'Sincerity Lacking in VCTs', 'The Big Issue: Panic over HIV testing'; 'Fix it now, VCT debate getting out of control' and 'Government defends VCTs' (Figure 2). The debate was intended to air the concerns of the Association about the professional erosion of the laboratory staff but actually gave a platform for donors to sponsor articles explaining quality assurance procedures and stimulated public interest in VCT sites.

Despite this the conflict was barely noticed outside Nairobi. In fact some of the district and provincial interviewees thought the bad publicity may have stimulated interest in VCT. Most had good working relationships between the routine laboratory staff and VCT.

"At national level there has been a lot of politics but in our area so far no problem." (District Medical Officer of Health, KII.)

The laboratory members interviewed would have welcomed their inclusion in planning, in study design to evaluate quality, and in early stages of roll-out. The omission of the official representatives of the professional association and senior laboratory personnel working with the government was felt to be an error. However, there were still those who felt that an early official lab presence may have inhibited scale up in the public sector.

"If they were on the taskforce earlier it might have affected scale up. Depending on how strong they were or how forceful of their point of view it could have blocked expansion." (Female, donor, KII.)

### Content of task shifting

The initial strategies for VCT scale up did not make task shifting policies for VCT explicit. Assumptions were made that health care workers, taken from a number of different cadres, would perform dual roles.

"VCT are manned from all directions. Some are public health technicians, some are nurses etc." (Male, programme officer, KII.)

There was an awareness that similar HIV counselling programmes had failed in the past due to the impact of dual roles (doing counselling as well as nursing) on provider stress, high attrition rates and inconsistent service delivery. This led to deliberate policies on the prevention of counsellor burn out through a policy of mandatory attendance at support supervision sessions. Furthermore lay personnel and 'volunteer counsellors' were encouraged to apply for training positions and to provide services with NGO support. Despite efforts to mitigate the impact of task shifting on strained resources, concerns arose not only about the lack of deliberate policy but also about the lack of clear line management structures that ensued. Staff reported to - and were evaluated by - their former bosses in their main discipline, many of whom were not versed in the expectations or job descriptions for VCT counsellors. This created tensions. For example, counsellors were told to see a max of 8-15 clients per day. A departmental head who had over 50 patients waiting to be served in another department by one nurse/midwife could not understand why someone would serve only 8 or 15 clients. Those who tried to perform both roles were frustrated and overworked. This resulted in internal conflict or frustrated clients as VCT service opening times became irregular in order to fit round other work expectations.

Subsequently the lack of promotional structures and of recognition for counselling as a cadre threatened the task shifting policies as far as the use of other health professionals was concerned and increasingly opened the door for lay counsellors to provide services in health facilities.

On the other hand, policies around quality assurance were integral to the scale-up strategy in Kenya [18] and were central in guiding how to task shift to lay counsellors, many of whom were volunteers. The guidelines and training curriculum became a key element of the subsequent site registration and quality assurance systems. Within a year of the establishment of the taskforce, the guidelines had been disseminated throughout Kenya and an enforced registration system meant that all VCT sites should be operating under the same standards *(Report on piloting of registration system VCT taskforce minutes Nov 7th 2001)*. Only registered sites could access the free HIV test kits funded by a World Bank loan [41] in exchange for data on client uptake. A national data form was developed and registered sites completed a form for each individual.

"The government took a loan from the World Bank for the purchase of test kits. It helped us. Free kits probably accelerated the process and allowed us to control it." (Male MoH taskforce member, KII.)

The registration system then formed the basis of a comprehensive quality assurance programme that included supervision of counselling and testing, continuing education, laboratory-based testing using dried blood spots and inspection of sites. In late 2003 a National Quality Assurance Team was established, including laboratory supervisory staff, who visited all sites annually. The team found high standards of testing in registered sites [40] and reported high client confidence in the approach used.

"It is important for the client and the counsellor to do their thing together and for the client to watch. Then clients have absolute confidence in their results." (Male, laboratory, KII.)

## Discussion

Our findings indicate that programmers have to deal with more than the content (the technical components) of a policy in order to get it successfully implemented. A qualitative approach has revealed multiple complex layers as individuals and groups with their own personal and political histories and interactions all play a role. Our methodology and our policy analysis, that was not restricted to the policy content, but included all four components of the policy triangle, have enabled an in-depth understanding of the human resource implications of VCT scale up from the perspective of the full breadth of task force members, including representative, experienced service providers. First we need to be clear about the actors and their interests (which may or may not be supportive). Next we need to understand the context as this might influence the interests of the actors (for example an employment embargo heightening the opposition of the lab staff to task-shifting). Finally, with a better understanding of the actors and the context and the potential risks involved, the content and the process can be better planned. These findings have resonance for the provision of new services, such as new rapid diagnostic tests or new care pathways in many settings.

Despite staffing shortages, medical training colleges in many resource-poor countries continue to produce health care workers, without significant overlapping skills, who strive to maintain high standards. These health care workers are understandably protective of professional boundaries and this protectionism has the potential to threaten or even deliberately sabotage the move to task shifting. The advent of a new testing technology which could be performed without extensive laboratory training clearly raised concerns among laboratory workers Kenya. The VCT taskforce compounded this by failing to engage adequately with all the officially recognized groups (Board and Association) of laboratory seniors. A better understanding of the preceding political conflicts between the Association and the Board as well as of the positions and legitimate concerns of the members would have allowed a smoother path to scale up. Some members' concerns may have been rooted in their training. As a result, despite documented high standards of lay counsellors conducting testing [40], the professional associations diverted time and attention away from supervision and the establishment of comprehensive quality assurance systems for HIV testing in all settings (including provider-initiated HIV testing and counselling) to do battle with VCT. Time has shown that their concerns over quality of testing are justified in the absence of comprehensive and funded systems of training, certification, data recording and external retesting of samples.

In keeping with findings from Zambia and Cameroon we highlight the importance of clearly re-defining roles and task-shifting procedures [6,42]. Our data also indicated that the success of task shifting in Kenya was in part due to the quality assurance systems for HIV testing. While the introduction of task shifting for HIV counselling and testing in Kenya has enabled rapid scale up of services, it was the flexibility around who conducts testing that allowed expansion into non-medical areas, outreach services and non-government facilities. This flexibility was possible through the scale up of one unified regulatory and quality assurance system [18]. Quality assurance systems for VCT scale up in Kenya are thus both part of the context (as they were planned as part of scale up from the outset) and the content as policies evolved and new systems piloted as scale up progressed.

In 2005 Kenyan unified guidelines broadened the scope of HIV testing further to include all health cadres with minimal training [43]. Here, even more so than in VCT, rapid expansion has threatened the quality of services, both in Kenya and globally [44,45]. External quality assurance systems for testing and counselling are not maintained in provider-initiated HIV testing and counselling to the same standard as those in VCT sites. Testing sites do not conduct proficiency panels and do not send dried blood spots from rapid tests to reference laboratories (*personal communication, NASCOP, 2008*). Indeed there is no national picture of where testing is conducted, who is doing the testing and what the quality of ward and clinic-based testing is. It has also become the expectation that every health care worker will do counselling in every clinical setting. While this has increased the rates of HIV testing and counselling, it has also led to missed opportunities for prevention messages and created a coercive atmosphere around testing in some settings [44,46]. Involving lay counsellors in busy clinics conducting HTC has been a successful approach in Botswana [47] and may yet have a role to play in PITC scale up with a renewed focus on universal access to HTC. Issues of sustainability, quality assurance of testing and the use of lay counsellors are still debated and local actors, processes and content are vital components in understanding how these opportunities can be maximised in different contexts.

## Conclusion

Our findings are still relevant today as new approaches to HTC are scaled up within health systems and staff struggle to balance the human resources requirements with the need to maintain quality of service and laboratory and counselling professionalism. Our analysis shows that the use of lay counsellors and health care workers from a number of cadres to conduct counselling as well as testing in Kenya served to strengthen rather than undermine primary care without diverting significant resources from laboratories or depleting any one cadre. Concentrating only on the content or technical side of programme scale up is likely to fail, whatever the approach to HTC scale up that is being used. A critical and informed analysis of context and actors and the prioritisation of quality assurance are central to the successful scale up of HIV services.

## Conflict of interests

The authors declare that they have no competing interests.

## Authors' contributions

MT conceived and designed the study, conducted the interviews, analysed the data and wrote the paper. ST designed the study, reviewed data analysis and critically appraised the document. DGL contributed to the analysis and read and critically reviewed the document. AI, JHN, CW and J were intimately involved in the scale up of VCT that the paper describes and read and critically appraised the document. TM contributed to the writing of the paper and read and critically appraised later versions. All authors have read and approved the final version of this manuscript.

## Pre-publication history

The pre-publication history for this paper can be accessed here:

http://www.biomedcentral.com/1471-2458/11/812/prepub
